# Growth kinetics, fatty acid composition and metabolic activity changes of *Crypthecodinium cohnii* under different nitrogen source and concentration

**DOI:** 10.1186/s13568-017-0384-3

**Published:** 2017-04-20

**Authors:** Waseem Safdar, Muhammad Shamoon, Xinyi Zan, Junaid Haider, Hafiz Rizwan Sharif, Muhammad Shoaib, Yuanda Song

**Affiliations:** 1 State Key Laboratory of Food Science & Technology, School of Food Science & Technology, Jiangnan University, Wuxi, 214122 Jiangsu People’s Republic of China; 2 Colin Ratledge Center for Microbial Lipids, School of Agriculture Engineering and Food Science, Shandong University of Technology, Zibo, 255049 Shandong People’s Republic of China

**Keywords:** *Crypthecodinium cohnii*, N-sources, lipid accumulation, DHA, G6PDH, ATP:citrate lyase

## Abstract

The effect of varying concentrations of the nitrogen source on the growth kinetics, lipid accumulation, lipid and DHA productivity, and fatty acid composition of *C. cohnii* was elucidated. Growth of *C. cohnii* was in three distinct growth stages: cell growth, lipid accumulation and a final lipid turnover stage. Most of lipids were accumulated in lipid accumulation stage (48–120 h) though, slow growth rate was observed during this stage. NaNO_3_ supported significantly higher lipid content (26.9% of DCW), DHA content (0.99 g/L) and DHA yield (44.2 mg/g glucose) which were 2.5 to 3.3-folds higher than other N-sources. The maximum level of C16–C18 content (% TFA) was calculated as 43, 54 and 43% in lipid accumulation stage under low nitrogen (LN, 0.2 g/L), medium nitrogen (MN, 0.8 g/L) and high nitrogen (HN, 1.6 g/L) treatments, respectively. Cultures with LN, by down-regulating cell metabolism, trigger onset of lipogenic enzymes. Conversely, NAD^+^/NADP^+^-dependent isocitrate dehydrogenase (NAD^+^/NADP^+^-ICDH) were less active in LN than HN treatments which resulted in retardation of Kreb’s Cycle and thereby divert citrate into cytoplasm as substrate for ATP-citrate lyase (ACL). Thereby, ACL and fatty acid synthase (FAS) were most active in lipid accumulation stage at LN treatments. Glucose-6-phosphate dehydrogenase (G6PDH) was more active than malic enzyme (ME) in lipid accumulation stage and showed higher activities in NaNO_3_ than other N-sources. This represents that G6PDH contributes more NADPH than ME in *C. cohnii*. However, G6PDH and ME together seems to play a dual role in offering NADPH for lipid biosynthesis. This concept of ME together with G6PD in offering NADPH for lipogenesis might be novel in this alga and needed to be explored.

## Introduction

*Crypthecodinium cohnii* is a heterotrophic dinoflagellate that has been used for the commercial production of oil rich in docosahexaenoic acid (DHA, 22:6 n-3) since mid 1990s. DHA-rich oil form *C. cohnii* is simple in Fatty acid profile, cost effective and without typical fishy smell (Wynn et al. 2005). Many of studies were reported evaluating the effect of nutrients and factors such as glucose, carbon, culture temperature, salinity and light etc. for lipid production in *C. cohnii* (DeSwaaf et al. 2003; Gong et al. 2015; Liu et al. 2015; Liu et al. 2016a, b; Pleissner and Eriksen 2012; Ratledge et al. 2001; Silva et al. 2016; Sun et al. 2017). In resent researches, the effect of N has been positively exploited in *Monoraphidium* sp. (Dhup and Dhawan 2014), *Scenedesmus abundans* (González-Garcinuño et al. 2014), *Saccharomyces cerevisiae* (Portugal-Nunes et al. 2017), *S. rubescens* (Lin and Lin 2011) and *Stigeoclonium* sp. (Liu et al. 2016a, b) for the production of commercially important lipids.

As N plays a pivotal role for the synthesis of both protein and nucleic acid and an increased in N supply profoundly enhanced their production which, otherwise, ceased when culture becomes N-limited. This directly relates with biomass reduction and hence, increases lipids (Ratledge and Wynn 2002). Batch fermentation methodologies offer essential experimental settings and result in lowered biomass yield, facilitating the higher lipid accumulation via maintaining enclosed N-starved environment. N-limitation in culture condition stimulated lipid accumulation in oleaginous microorganisms (Ratledge 2014; Zhao et al. 2015). It is also widely accepted that most oleaginous microorganisms start accumulating lipids in presence of excess carbon and limited N-sources in the medium (Ikaran et al. 2015; Lv et al. 2010; Ördög et al. 2016). However, the final algal biomass production typically depends on strain capacity and fermentation strategy which may exceed as high as 100 g/L DCW in ideal conditions (Gaffney et al. 2014).

Astoundingly, there is only one report about glutamic acid (as N-source) limitation cause reduction in growth with no significant effect on specific lipid contents of *C. cohnii* CCMP 316 (Pleissner and Eriksen 2012). To our knowledge, there is no other experimental evidence elaborating the influence of other N-sources (nitrates and ammonium) on cell growth and lipid accumulation in *C. cohnii*. Therefore, present study was aimed to explore the effects of different N-sources and concentrations on biochemical and physiological changes in *C. cohnii*. Furthermore, the role of N influence on metabolic activities of key enzymes; fatty acid synthase (FAS), malic enzyme (ME), ATP citrate lyase (ACL), glucose 6-phosphate dehydrogenase (G6PDH), citrate synthase (CS), NADP^+^-dependent isocitrate dehydrogenase (NADP^+^-ICDH) and NAD^+^-dependent isocitrate dehydrogenase (NAD^+^-ICDH) was also elucidated in regulating the lipid accumulation. Our work provides comprehensive understanding of lipid accumulation and new insights which may prove promising for the effective lipid enhancement and particularly DHA production in *C. cohnii*.

## Materials and methods

### Microorganism and culture conditions

*Crypthecodinium cohnii* (ATCC 30555) was purchased from the America Type Culture Collection (ATCC) and maintained in sterilized ATCC460 medium. Batch cultures were performed in 5 L fermenters (NBS Bioflo 115, USA) with 10% (v/v) inoculum size and 3 L working volume. The inocula were grown in ATCC 460 A2E6 medium for three days in a 500 mL flask before centrifugation and re-suspension in optimized experimental medium composed of (g/L): NaCl, 23.5; Na_2_SO_4_, 3.9; NaHCO_3_, 0.2; MgCl_2_·6H_2_O, 10.6; KCl, 0.7; CaCl_2_, 1.1; KBr, 0.1; glucose, 27; disodium glycerophosphate, 15; glutamic acid, 0.2; tris, 3; SrCl_2_·6H_2_O, 0.04; K_2_HPO_4_, 0.1; 5.0 mL of metal mixture (g/L): (Na_2_EDTA, 10; FeCl_3_·6H_2_O, 0.5; H_3_BO_3_, 10; MnCl_2_·4H_2_O, 1.6; CoCl_2_·6H_2_O, 0.005); ZnCl_2_, 0.1; 1.0 mL vitamin solution (mg/L): (Biotin, 3; Thiamine, 1000), and N-source. Different concentrations (indicated in the text) of (NH_4_)_2_SO_4_, (NH_2_)_2_CO, NH_4_HCO_3_ and NaNO_3_ were used as N-sources.

Initial pH was adjusted to 6.5 and controlled at this value by automatic addition of 2 M HCl and 2 M KOH. Stepwise dissolved oxygen tension was achieved by shifting agitation speed from 700 to 300 rpm. Foaming was controlled by automatic addition of 5% (w/v) silicone SE-2. The medium inside the fermenters were sterilized by autoclaving at 121 °C for 20 min. All fine chemicals were purchased from Sigma-Aldrich unless otherwise. Three replicates were performed in all experiments.

### Determination of physiological parameters

Biomass concentration, from daily harvested 50–100 mL samples, was determined gravimetrically by centrifugation (10 min, 3000×*g* for 5 °C). Briefly, the cell pellet was rinsed twice with distilled water, frozen overnight at −80 °C and weighted following the lyophilisation for 24 h. Biomass concentration was expressed as dry cell weight (DCW) per liter. The biomass productivity (*P*_DCW_) was calculated using Eq. 1.

1 $$P_{\text{DCW}} \left( {{\text{g}}/{\text{L}}\;{\text{day}}} \right) = \frac{{{\text{DCW}}_{\text{f}} - {\text{DCW}}_{\text{i}} }}{{{\text{T}}_{\text{f}} - {\text{T}}_{\text{i}} }}$$

where DCW_f_: final biomass content (g/L); T_f_: harvesting time (day); DCW_i_: initial biomass production (g/L); T_i_: cultivation time (day).

Indophenol method (Chaney and Marbach 1962) was used to determine ammonium concentration in the culture. Glucose concentration in the culture was measured using glucose oxidase Perid-test kit (Shanghai Rongsheng Biotech Co., Ltd). Soluble phosphate was determined using colorimetric method (Ren et al. 2013). Absorbance of the supernatant was measured at 885 nm, after proper dilution with deionized water. Phosphate concentration was determined by using a calibration curve made with KH_2_PO_4_ in the range 10–70 μM. Nitrate concentration in the culture medium was determined spectrophotometrically according to the method described by Collos et al. (1999). Briefly, culture samples were daily harvested, centrifuged (3000×*g*, 5 °C for 10 min) and supernatant was collected. The absorbance was measured at 220 nm after a proper dilution with deionized water (Ikaran et al. 2015). The absorbance values were converted to nitrate concentration using a standard calibration curve made with NaNO_3_ in the range 0–10 mM. As lipid accumulation completely ceased at N-source concentration above 20 mM, therefore treatment above this value was excluded from the analysis.

### Determination of fatty acid composition

Lipids were extracted by a modified protocol of Bligh and Dyer (1959) from freeze-dried cells. The lipid productivity (*P*_Lipid_) and DHA productivity (*P*_DHA_) were calculated by following formulae 2 and 3:

2 $$P_{\text{Lipid}} \left( {{\text{g}}/{\text{L}}\;{\text{day}}} \right) = \frac{{C_{\text{f}} \times {\text{ DCW}}_{\text{f}} - C_{\text{i}} \times {\text{ DCW}}_{\text{i}} }}{{{\text{T}}_{\text{f}} - {\text{ T}}_{\text{i}} }}$$

3 $$P_{\text{DHA}} \left( {{\text{g}}/{\text{L}}\;{\text{day}}} \right) = \frac{{C_{\text{DHA}} \left( {{\text{g}}/{\text{g TL}}} \right) \times {\text{Lipid}}\left( {{\text{g}}/{\text{L}}} \right)}}{{{\text{Time }}\left( {\text{day}} \right)}}$$

where C_f_: final lipid content (g/L); C_i_: initial lipid content; TL: total lipid.

For fatty acid (FA) analysis, ~100 mg of lyophilized algal biomass was re-suspended in 5 mL chloroform: methanol (2:1 v/v) containing pentadecanoic acid (15:0, 2.0 mg/mL; Sigma) as an internal standard and 0.5 mg/mL butylated hydroxytoluene (BHT) as an antioxidant at room temperature for 24 h. After centrifugation (5 min, 3000×*g*), the supernatant containing extracted lipids were transferred into a clean tube and residue was re-suspended in chloroform: methanol (2:1 v/v) at room temperature for 12 h. After centrifugation, supernatants from both extractions were combined and washed with 2 mL of saturated NaCl. The resultant fatty acid methyl esters (FAMEs) were extracted in hexane and analyzed by gas chromatography (GC-2010; Shimadzu Co., Kyoto, Japan). GC was equipped with a capillary DB-WAX column (30 m × 0.32 mm, ð 0.25 μm, Agilent, USA) and FID detector; helium was the carrier gas. The oven temperature was initially held at 120 °C for 3 min and raised to 180 °C by increasing 5 °C per min, then raised to 260 °C at the rate 5 °C per min, and finally held at 260 °C for 5 min. The FAs were identified with standards (Sigma, USA). Three biological replicates were performed in all experiments and analyzed using a one-way analysis of variance (ANOVA) analysis using SPSS Statistics 19.

### Determination of enzyme activities

#### Preparation of cell-free enzyme extracts

Biomass, periodically harvested by centrifugation from the fermenters, was washed twice with washing buffer (200 mM Tris/HCl, pH 7.4, 2 mM DTT and 1 mM EDTA) and re-suspended in the same buffer. After being ultrasonically (Scientz-II D sonifier) disrupted at 225 × 4 s with cooling in between on ice for 15 min, the cell suspensions were centrifuged (10,000×*g*; 10 min at 4 °C). The supernatant containing cytoplasmic and mitochondrial enzymes was subjected to enzyme activity analysis. Standard Bradford method was used to determine the protein concentration.

#### Enzyme activity analysis

Activities of fatty acid synthase (FAS), malic enzyme (ME), ATP:citrate lyase (ACL), glucose-6-phosphate dehydrogenase (G6PDH), citrate synthase (CS), NADP^+^-dependent isocitrate dehydrogenase (NADP^+^-ICDH) and NAD^+^-dependent isocitrate dehydrogenase (NAD^+^-ICDH) were determined in supernatant fraction by continuous spectrophotometric assays at 30 °C. For FAS analysis, the reaction mixture contained 0.3% (w/v) BSA, 4 mM DTT, 100 mM KH_2_PO_4_/KOH (pH 6.5), 0.18 mM acetyl-CoA, 2.5 mM EDTA, 0.09 mM malonyl-CoA, 0.14 mM NADPH and cell free extract. The reaction mixture for ME analysis contained 25 mM malate, 3 mM MgCl_2_, 80 mM KH_2_PO_4_/KOH (pH 7.5), 0.6 mM NADP^+^ and cell free extract; the reaction was initiated by adding 25 mM malate. For ACL analysis, the reaction mixture contained 0.3 mg CoA/mL, 10 mM sodium azide, 10 mM Tris/HCl (pH 8.6), 0.2 mM NADH, 10 mM mercaptoethanol, 5 units malate dehydrogenase/ml, 5 mM ATP (pH 7.5) and cell free extract. For G6PDH analysis, the reaction mixture contained 0.3 mM NADP^+^, 5 mM MgCl_2_, 50 mM Tris/HCl pH 8.0, 2.5 mM glucose 6-phosphate and cell free extract. For CS analysis, the reaction mixture contained cell free extract, 0.12 mM acetyl-CoA, 0.2 mM oxaloacetate, 400 mM Tris/HCl (pH 8.0) and 0.25 mM DTNB. NADP^+^-ICDH activity was assayed as described by Wynn et al. (1999) and NAD^+^-ICDH activity was assayed as described by Wynn et al. (2001). For ME, G6PD and CS an increase in OD was measured at 30 s. For ACL and FAS, a decrease in OD was measured at 30 s. An interval of 3 min at 340 nm was given for each enzyme. One unit of enzyme activity (U) was defined as “the amount of enzyme, required to produce 1 mol enzymatic reaction product in 1 min in the above mentioned conditions”. The protein concentration in the cell free extract was determined by standard Bradford method. Three biological replicates were used for each enzyme activity to assess reproducibility.

## Results

### Effect of different N-sources on *C. cohnii*

#### Growth kinetics and lipid accumulation

**Fig. 1:**
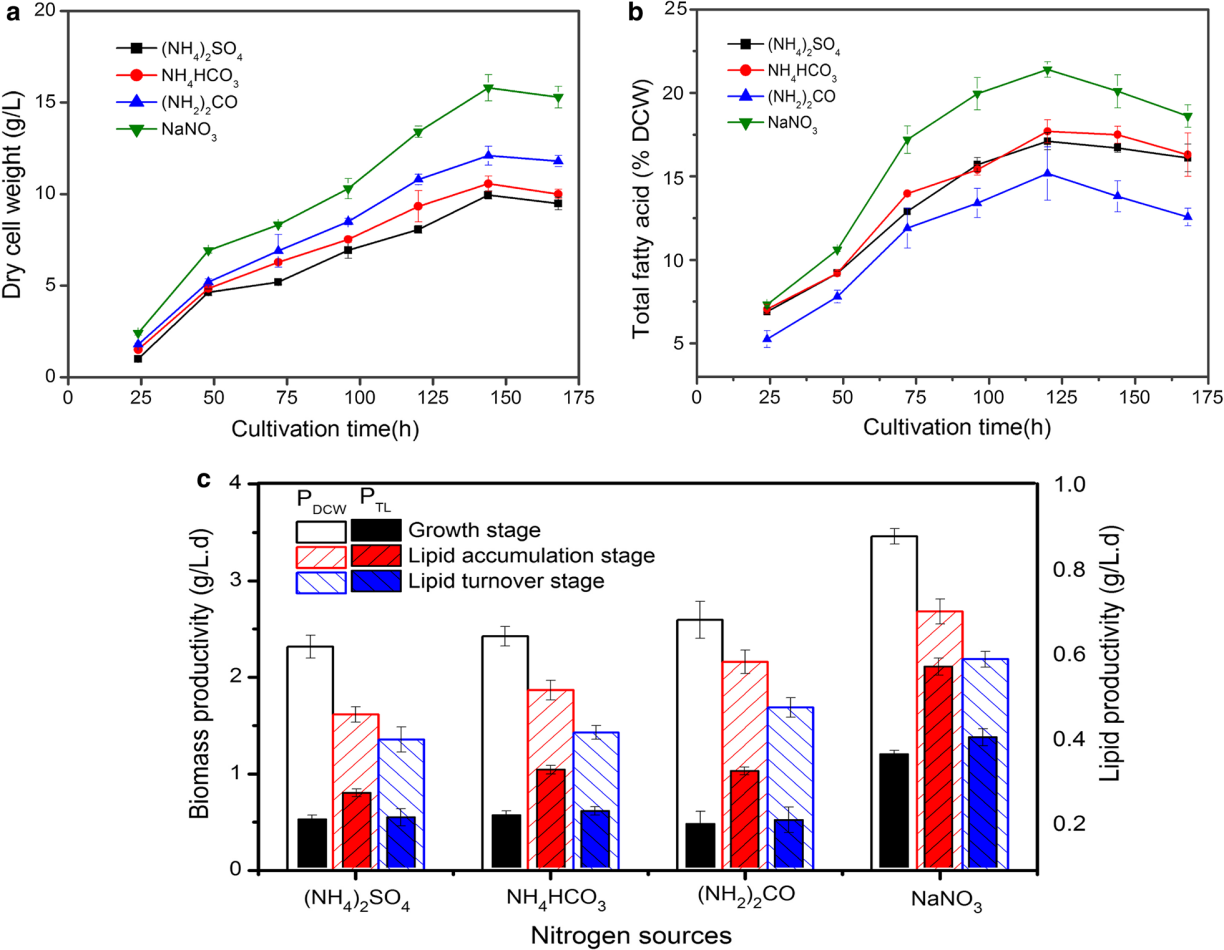
The time-course profile of cell growth and lipid accumulation in *C. Cohnii* under different N-sources. **a** Dry cell weight (DCW, g/L), **b** total fatty acid (TFA, % DCW) of *C. cohnii* for cultured for 7 days. **c** Biomass productivity (P_DCW_, g/L day) and lipid productivity (P_TL_, g/L day) of *C. Cohnii* grown on different N-sources at three different growth stages. All experiments were performed in triplicate. The data presented here is mean ± SD

The time-course profile of cell growth and lipid accumulation of *C. cohnii* cultured for 7 days under the influence of different N-sources is given in Fig. 1. A particular pattern of growth and lipid accumulation was observed in *C. cohnii* during the entire growth period. Rapid cell growth was observed from 0 to 48 h when there is a sufficient supply of N and glucose; this was identified as *‘‘cell growth stage”*. After 48 h, a continuous but slower growth was noticed throughout the remaining culture time (Fig. 1a). Compared to ammonium sulphate, ammonium bicarbonate and urea, the biomass (DCW) with sodium nitrate supplementation was significantly higher and reached up to the highest level of 15.82 ± 0.72 g/L at 144 h of cultivation. Simultaneously, ammonium sulphate and ammonium bicarbonate can support almost similar growth pattern of *C. cohnii* under the investigated conditions which is however lower than urea (Fig. 1a).

After 48 h, when cell growth slowed, lipids commence to accumulate till 120 h as long as there was sufficient carbon source (above 4.5 g/L) in culture mediums and identified as *‘‘Lipid accumulation stage”*. Maximum lipid content was obtained at 120 h of cultivation in all treatments. Highest level of total lipid (% DCW) was accumulated 21.4 ± 0.5% in NaNO_3_ treatment (Fig. 1b). In contrary with the biomass, urea showed the lowest lipid accumulation rate (10.7 mg/L h) as compared to (NH_4_)_2_SO_4_ (12.7 mg/L h) and NH_4_HCO_3_ (12.3 mg/L h) in lipid accumulation stage. Whereas, NaNO_3_ showed the highest lipid accumulation rate (21.73 mg/L h) which was 1.9 folds higher than that in the growth stage (11.3 mg/L h). After 120 h, lipid accumulation ceased and lipid turnover occurred from 120 to 168 h and identified as “*lipid turnover stage*’’. Similar trend of lipid accumulation was also observed in treatment with other N-sources. There was a significant difference in cell growth and lipid accumulation when cultured on different N-sources (*P* < 0.05). Along with biomass, lipid productivity is also important to evaluate the overall performance of microalgae. Maximum biomass productivity (3.5 g/L day) and lipid productivity (0.6 g/L day) was obtained with NaNO_3_ (Fig. 1c).

**Fig. 2:**
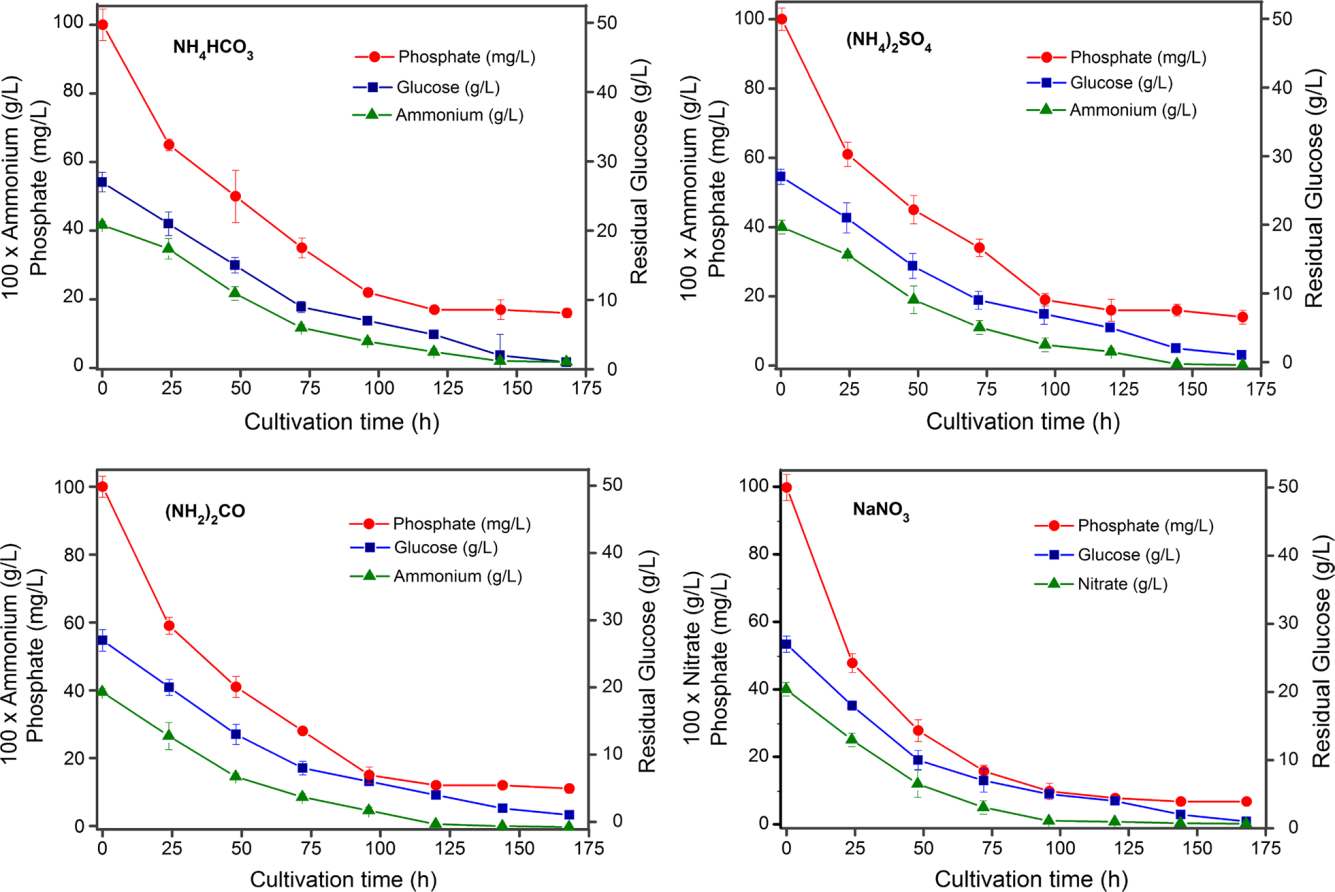
The time-course profile of residual substrates (glucose, nitrate and phosphorus) concentration in *C. cohnii* cultured on different nitrogen sources for 7 days. All experiments were performed in triplicate. The data presented here is mean ± SD

Figure 2 shows the time-course profile of substrates depletion (glucose, nitrate and phosphorus) by *C. cohnii*. As expected, the uptake rate of glucose and nitrate in NaNO_3_ supplemented medium was significantly higher than others. However, there was no significant difference in phosphorus assimilation in the cultures treated with different N-sources (*P* < 0.05). Conversely, the assimilation rate of glucose in NaNO_3_ supplemented medium gradually decreased from cell growth stage (0.36 ± 0.05 g/L h) to lipid accumulation (0.22 ± 0.09 g/L h) and followed by lipid turnover stage (0.14 ± 0.06 g/L h). Similar trend was observed with nitrate and phosphate (Fig. 2). Lipid yield at the cost of glucose assimilated (mg/g GLC) was also calculated (Table 1). Results showed that highest lipid yield was obtained when grown on NaNO_3_ (130.3 ± 4.5 mg/g GLC) in lipid accumulation stage which was 3.4 folds higher than that in growth stage.

**Table 1:** Comparison of DHA content, DHA productivity, lipid and DHA yield of *C. cohnii* under different N-sources

N-source	Growth stage	C_DHA_ (g/L)	P_DHA_ (mg/L day)	Y_L_^a^ (mg/g GLC)	Y_DHA_^a^ (mg/g GLC)
(NH_4_)_2_SO_4_	Growth	0.05 ± 0.0	22.4 ± 4.9	35.5 ± 2.2	3.7 ± 0.7
	Lipid accumulation	0.35 ± 0.1	70.9 ± 8.9	65.6 ± 1.4	16.9 ± 2.5
	Lipid turnover	0.42 ± 0.1	60.3 ± 7.1	71.3 ± 5.3	19.7 ± 1.5
NH_4_HCO_3_	Growth	0.05 ± 0.0	25.8 ± 1.6	28.9 ± 4.2	3.9 ± 1.2
	Lipid accumulation	0.39 ± 0.08	77.3 ± 3.9	74.4 ± 1.1	17.6 ± 0.4
	Lipid turnover	0.43 ± 0.05	60.7 ± 4.4	66.8 ± 3.5	19.1 ± 1.5
(NH_2_)_2_CO	Growth	0.06 ± 0.0	27.5 ± 3.1	34.3 ± 6.9	4.2 ± 1.5
	Lipid accumulation	0.46 ± 0.05	91.1 ± 3.2	75.1 ± 2.2	20.7 ± 0.6
	Lipid turnover	0.46 ± 0.11	65.3 ± 4.2	73.4 ± 3.4	20.6 ± 0.9
NaNO_3_	Growth	0.13 ± 0.02	63.4 ± 1.6	43.1 ± 3.5	7.4 ± 1.4
	Lipid accumulation	0.97 ± 0.12	193.8 ± 2.8	130.3 ± 4.5	44.1 ± 3.6
	Lipid turnover	0.99 ± 0.05	141.1 ± 5.7	127.1 ± 9.6	44.1 ± 2.4

All experiments were performed in triplicate. The data presented here is mean ± SD

*C*_*DHA*_ DHA content, *P*_*DHA*_ DHA productivity (g/L day), *Y*_*L*_ lipid yield (mg/g glucose), *Y*_*DHA*_ DHA yield (g/g glucose)

^a^ Yield was calculated on basis of glucose utilized

#### Fatty acid shifts in the three growth stages

**Fig. 3:**
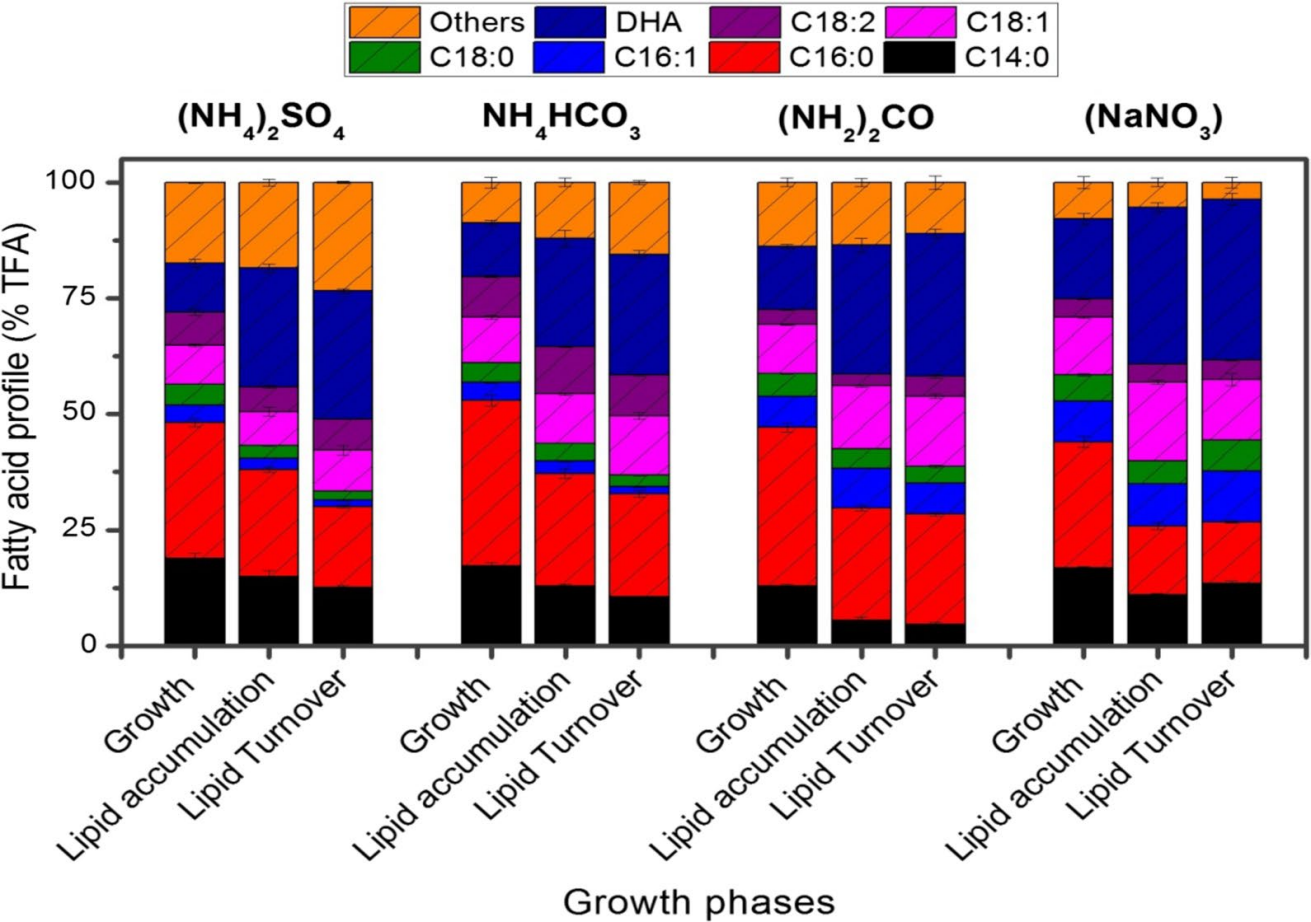
Fatty acid profile (% total fatty acid) of *C. Cohnii* under different N-sources in three growth stages; cell growth, lipid accumulation and lipid turnover stage, harvested on 48, 120 and 168 h, respectively. All experiments were performed in triplicate. The data presented here is mean ± SD

Fatty acid profile was presented in three growth stages of microalgae under the influence of different N-sources (Fig. 3). Regarding the fatty acid composition, one of the primary goals of this study was to increase DHA (C22:6n3) content in *C. cohnii*. The highest DHA content was attained in lipid turnover stage (35.7 ± 1.3% TFA) with a slight difference from lipid accumulation stage (34.8 ± 0.9% TFA) under NaNO_3_ which was 32–44% higher than that of NH_4_HCO_3_ (Table 1). Similar results were found in newly isolated *Crypthecodinium* sp. SUN, which yielded 34.1 ± 0.3% TFA at 120 h and 35.0 ± 0.2% TFA at 144 h under light (Sun et al. 2017). For comparison, the overall DHA productivity, as well as yield (per gram of glucose exhaustion) during three growth stages is given in Table 1. It was obvious that NaNO_3_ supported significantly higher DHA content (0.99 ± 0.05 g/L), DHA productivity (193.84 ± 2.76 mg/L day) and DHA yield (44.16 ± 3.64 mg/g glucose) which were 2.1 to 2.8-folds higher than other N-sources (*P* < *0.001*).

Compared to DHA, in growth stage palmitic acid (C16:0) was produced at the highest percentage (27.2–35.7% TFA) among all fatty acids in all treatments. A gradual decline was observed in saturated fatty acids (SFAs) myristic acid (C14:0), C16:0 and stearic acid (C18:0) in all the four treatments throughout the cultivation time (Fig. 3). In contrast, mono-unsaturated fatty acids (MUFAs) palmitoleic acid (C16:1) and oleic acid (C18:1), increased during lipid accumulation stage and again decreased in lipid turnover stage in all treatments while no significant change was observed in Linoleic acid (C18:2n6) content (% TFA) (Fig. 3). Combined C16–C18 content (% TFA) was calculated as 53.1, 62.5, 59.7, 58.1% in cell growth stage and 51.9, 54.7, 53.1, 49.8% in lipid accumulation stage under (NH_4_)_2_SO_4_, NH_4_HCO_3_, (NH_2_)_2_CO, NaNO_3_ treatments, respectively (Fig. 3). Apparently, there is no significant difference in C16–C18 content in lipid accumulation stage under different N-sources; nevertheless, lowest C16–C18 content under NaNO_3_ might be due to highest DHA production. Presence of more than 50–60% of C16–C18 content in TFA suggested that *C. cohnii* can be considered as potential source for biodiesel production (Fig. 3).

### Effect of different nitrogen concentration

#### Growth kinetics and lipid accumulation

**Fig. 4:**
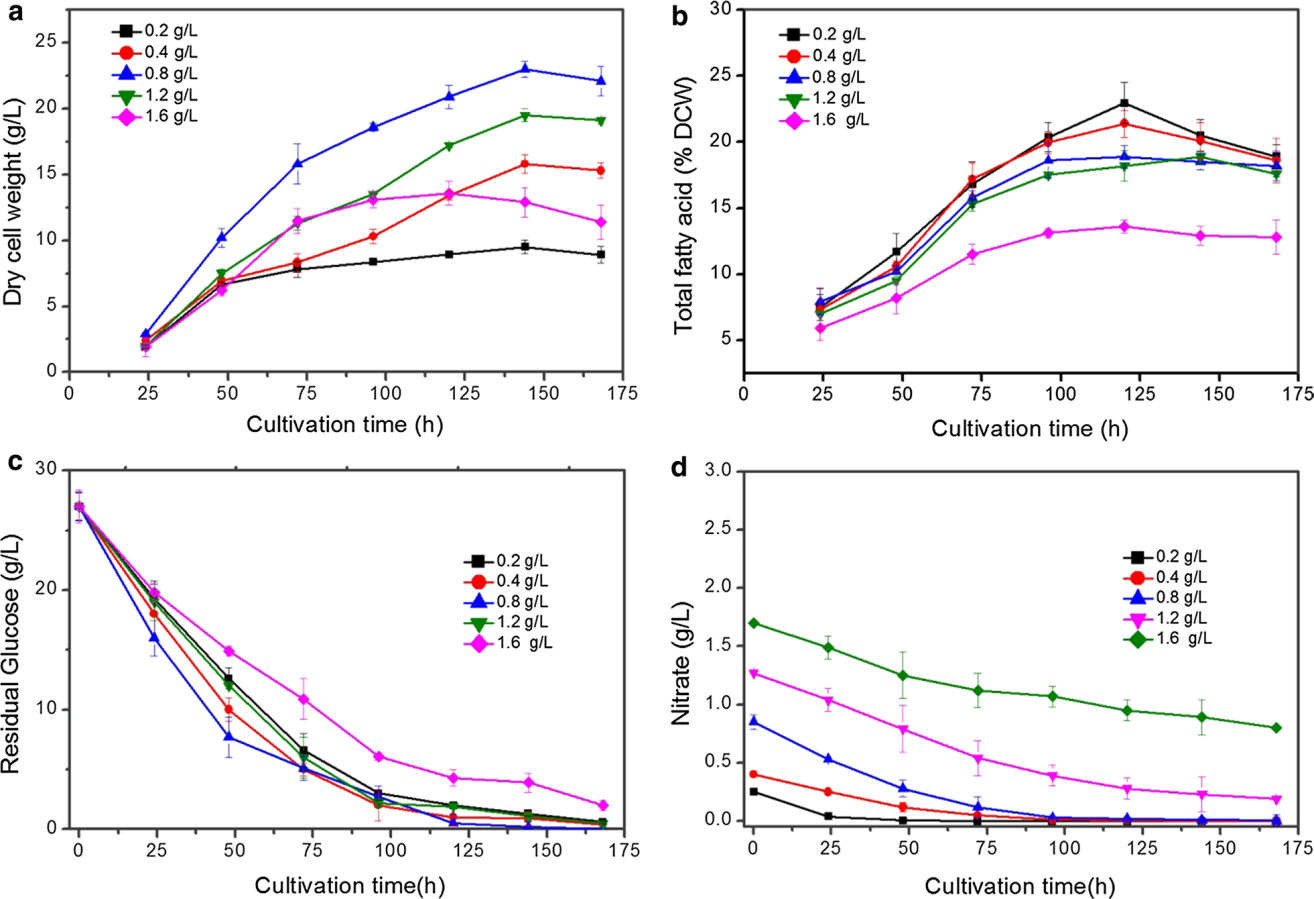
Comparison of biomass and lipid content of *C. cohnii* cultured on different NaNO_3_ concentrations. **a** The time-course profile of dry cell weight (DCW), **b** total fatty acid (TFA, % DCW), **c** residual glucose (GLC) concentration and **d** residual nitrate concentration grown on different concentration of NaNO_3_ for 7 days. All experiments were performed in triplicate. The data presented here is mean ± SD

To investigate an optimal N-supply for growth and lipid accumulation of *C. cohnii*, different concentrations (0.2, 0.4, 0.8, 1.2, 1.6 g/L) of NaNO_3_ supplementation were scrutinized. Figure 4 illustrates the effect of different concentrations of NaNO_3_ on dry cell weight (DCW) and lipid accumulation. Rapid cell growth was observed from 0 to 48 h when there is a sufficient supply of N and glucose (Fig. 4a). Comparable biomass yield (0.3 g/g glucose) was obtained at 24 h of cultivation in all NaNO_3_ treatments. After 36 h N was completely consumed in the control culture supplemented with 0.2 g/L NaNO_3_ and growth was restricted. Consequently, during the remaining 5 days, cells were N-starved (Fig. 4d). In contrast, cells grown on other concentrations (0.4, 0.8, 1.2, 1.6 g/L) of NaNO_3_ displayed continuous but slower growth throughout the remaining culture time. From Fig. 4a and d, it is noticeably obvious that algal growth was linearly correlated with N supply to a certain level and then show negative impact (decline).

As shown in Fig. 4a, growth was robustly improved with increasing concentration from 0.2 to 1.2 g/L and beyond this a negative impact on cell growth was noticed. The final biomass content at high nitrogen (HN, 1.6 g/L) concentration was 83.7% lesser than that of medium nitrogen (MN, 0.8 g/L) supplemented culture. Both, the biomass concentration (23.7 ± 0.61 g/L) and biomass productivity (5.10 ± 0.19 g/L day) reached the highest values with 0.8 g/L NaNO_3_ treatment (Fig. 4a; Table 2). The highest growth rate was also obtained when cultured on 0.8 g/L NaNO_3_ (212 mg/L h) which was 53% higher than that from low nitrogen (LN, 0.2 g/L) at 48 h of cultivation.

**Table 2:** Comparison of biomass yield, DHA content, DHA productivity, lipid and DHA yield of *C. cohnii* under different concentrations of NaNO_3_

NaNO_3_ conc.	Time	Y_DCW_^a^ (g/g GLC)	P_L_ (g/L day)	Y_L_^a^ (mg/g GLC)	C_DHA_ (g/L)	P_DHA_ (g/L day)	Y_DHA_^a^ (mg/g GLC)
0.2 g/L	Growth	0.46 ± 0.032	0.38 ± 0.009	54.05 ± 3.1	0.13 ± 0.004	0.064 ± 0.000	9.01 ± 0.09
	Lipid accumulation	0.47 ± 0.05	0.48 ± 0.01	126.76 ± 17.9	0.91 ± 0.008	0.18 ± 0.001	47.83 ± 2.19
	Lipid turnover	0.43 ± 0.063	0.28 ± 0.02	95.54 ± 14.9	0.78 ± 0.009	0.11 ± 0.001	38.12 ± 4.72
0.4 g/L	Growth	0.40 ± 0.007	0.37 ± 0.06	43.14 ± 8.98	0.13 ± 0.002	0.063 ± .002	7.46 ± 1.99
	Lipid accumulation	0.60 ± 0.009	0.57 ± 0.03	130.35 ± 21.6	0.96 ± 0.088	0.194 ± 0.004	44.05 ± 11.5
	Lipid turnover	0.68 ± 0.03	0.41 ± 0.01	127.11 ± 19.5	0.99 ± 0.08	0.141 ± 0.006	44.10 ± 8.55
0.8 g/L	Growth	0.52 ± 0.008	0.57 ± 0.02	53.90 ± 7.25	0.18 ± 0.004	0.089 ± 0.04	9.27 ± 1.24
	Lipid accumulation	0.92 ± 0.01	0.79 ± 0.002	175.56 ± 21.9	1.18 ± 0.01	0.236 ± 0.002	52.49 ± 5.52
	Lipid turnover	0.96 ± 0.04	0.62 ± 0.006	174.88 ± 14.9	1.29 ± 0.02	0.181 ± 0.001	56.14 ± 2.16
1.2 g/L	Growth	0.5 ± 0.008	0.35 ± 0.06	47.5 ± 4.69	0.11 ± 0.04	0.055 ± 0.000	7.36 ± 1.25
	Lipid accumulation	0.85 ± 0.057	0.63 ± 0.008	155.74 ± 8.55	0.66 ± 0.03	0.132 ± 0.007	33.01 ± 7.27
	Lipid turnover	0.87 ± 0.061	0.48 ± 0.02	154.2 ± 5.92	0.72 ± 0.006	0.103 ± 0.002	33.30 ± 9.68
1.6 g/L	Growth	0.51 ± 0.024	0.25 ± 0.004	42.01 ± 7.91	0.08 ± 0.001	0.038 ± 0.000	6.38 ± 165
	Lipid accumulation	0.76 ± 0.084	0.36 ± 0.009	104.5 ± 13.62	0.33 ± 0.002	0.065 ± 0.000	18.39 ± 3.52
	Lipid turnover	0.61 ± 0.062	0.21 ± 0.03	78.87 ± 8.46	0.26 ± 0.06	0.037 ± 0.005	14.19 ± 2.88

All experiments were performed in triplicate. The data presented here is mean ± SD

*Y*_*DCW*_ growth yield (g/g glucose), *P*_*L*_ lipid productivity (g/L day), *Y*_*L*_ lipid yield (mg/g glucose), *C*_*DHA*_ DHA content, *P*_*DHA*_ biomass productivity (g/L day), *Y*_*DHA*_ DHA yield (g/g glucose)

^a^ Yield calculated on basis of glucose utilized

In the culture, when cells were N-starved, a progressive increase in total fatty acid was observed from 48 to 120 h and reached up to 22.9 ± 1.6% of DCW which was almost similar to that of MN, however, 70% higher than that of HN supply (Fig. 4b). Indeed, N-starvation does not influence lipid production, however, maximum lipid content (4.3 ± 0.17 g/L), lipid productivity (0.79 ± 0.01 g/L day) and lipid yield (175.56 ± 21.9 mg/g glucose) was obtained with MN supplementation (Table 2). Time course profile of substrates depletion by *C. cohnii* at different concentrations of NaNO_3_ is given in Fig. 4c and d. All substrates (glucose, nitrate and phosphorus) were assimilated rapidly within 48 h after inoculation and then became slow gradually until the end of the culture period. Complimenting with growth, the uptake rate of glucose and nitrate in the MN medium was significantly higher than in HN and LN medium however, no significant difference was noticed in case of phosphate consumption (*P* < 0.05). In LN medium since protein biosynthesis was limited, this leads to restricted growth rate, however no significant effect on glucose utilization was observed (Fig. 4c). Highest yields of residual glucose 8.5 ± 0.9 g/L, phosphate 11.6 ± 1.3 mg/L and nitrate 0.8 ± 0.003 g/L was acquired in HN supplemented culture compared to phosphate 10.9 mg/L, glucose 2.5 ± 0.5 g/L and no detectable nitrogen in LN and MN cultures after 144 h (Fig. 4c, d).

#### Fatty acid composition

**Fig. 5:**
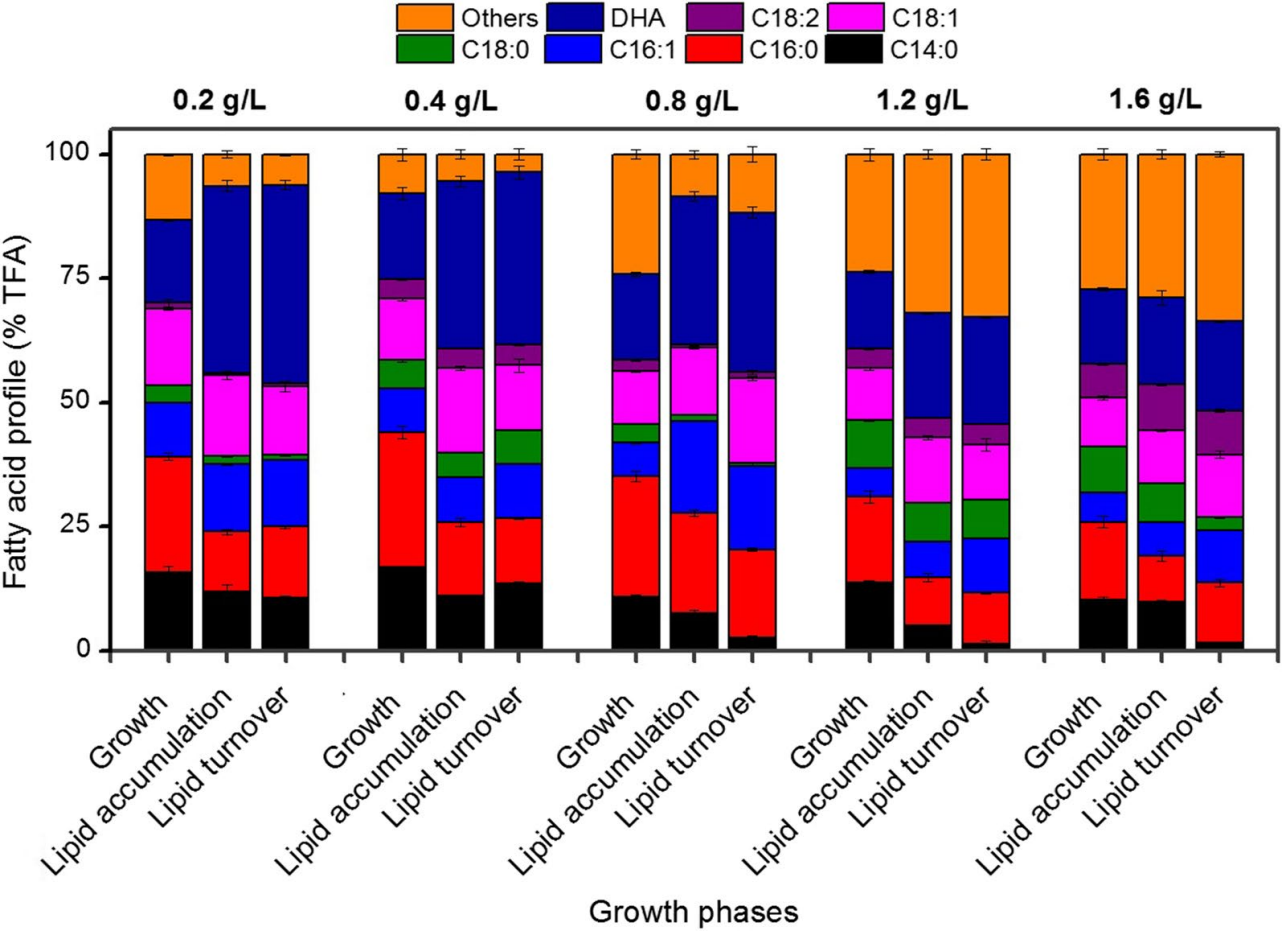
Fatty acid profile of *C. cohnii* under different NaNO_3_ concentration in three growth stages; cell growth, lipid accumulation and lipid turnover stage harvested on 48, 120 and 168 h, respectively. All experiments were performed in triplicate. The data presented here is mean ± SD

The fatty acids composition of *C. cohnii* under the influence of different NaNO_3_ concentrations is shown in Fig. 5. The predominant FA in all treatments was DHA (40.5 ± 0.52% TFA). In this study, DHA content was negatively influenced by NaNO_3_ concentrations. At HN treatment, DHA content was (18.5 ± 0.39% TFA) declined by 115% as compared to LN and MN treatment. Fatty acids present at moderate levels, as a percentage of the total FA, were C14:0 (16.8 ± 0.28%), C16:0 (27.21 ± 1.26%) and C18:1 (12.39 ± 0.27%) in MN treatment (Fig. 5). Despite of obtaining high levels of total fatty acids and DHA content at LN supplementation, biomass content was relatively low, which reduced the overall lipid productivity by 66% from MN treatment (Table 2). DHA yield was 32.9% lower in LN than HN supplementation (Table 2).

Saturated fatty acids (C14:0, C16:0 and C18:0) linearly decline from growth stage to lipid accumulation stage and then remained constant in lipid turnover stage in all N-treatments. In contrast, mono-unsaturated fatty acids (C16:1 and C18:1) increased during lipid accumulation stage and again slightly reduced in lipid turnover stage while polyunsaturated fatty acids robustly increased till end of the cultivation time. The maximum level of C16–C18 content (% TFA) was calculated as 43.88, 54.13 and 43.67% in lipid accumulation stage under LN, MN and HN treatments, respectively (Fig. 5). This highest C16–C18 content was obtained under MN.

#### Metabolic activity changes during lipid accumulation

**Fig. 6:**
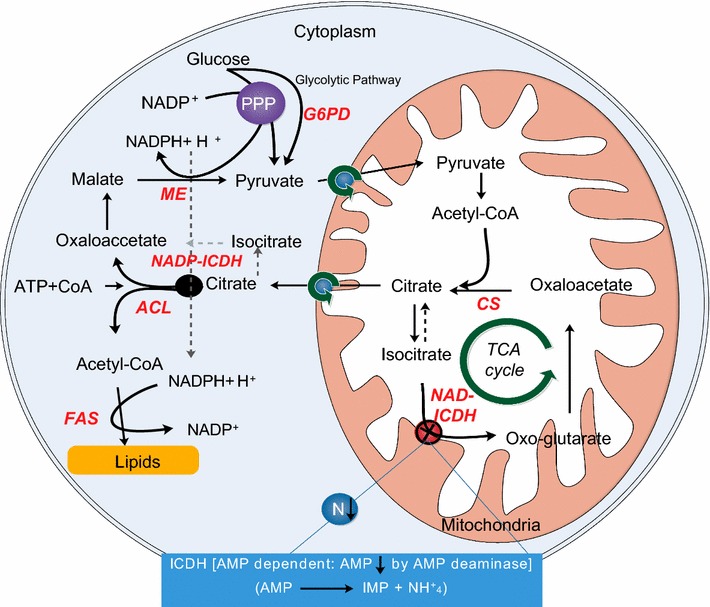
Metabolic pathway of lipid accumulation in oleaginous microorganisms and related key enzymes. *G6PD* glucose-6-phosphate dehydrogenase, *CS* citrate synthase, *NAD* ^*+*^ *-ICDH* NAD dependent isocitrate dehydrogenase, *NADP* ^*+*^ *-ICDH* NADP dependent isocitrate dehydrogenase, *ACL* ATP-citrate lyase, *ME* malic enzyme, *FAS* fatty acid synthase, *PPP* pentose phosphate pathway, *AMP* adenosine monophosphate, *IMP* inosine monophosphate

Figure 6 shows the metabolic pathway of fatty acid biosynthesis in oleaginous microorganisms. To compare the activity of key enzymes under N-sources during identified growth stages (growth stage, lipid accumulation stage and lipid turnover stage) samples were taken at 48, 120 and 168 h, respectively. The specific activities of FAS, ME, ACL G6PDH, NADP^+^-ICDH, CS and NAD^+^-ICDH were determined spectrophotometrically in each sample, the results are summarised in Tables 3 and 4. A gradual decline in the activities of G6PDH, ME, NADP^+^-ICDH, CS and NAD^+^-ICDH were detected from cell growth stage to lipid accumulation stage and followed to lipid turnover stage with lowest activities. Most probably this decline is triggered by substrate depletion particularly glucose in the culture. In contrast, FAS and ACL were the most active in lipid accumulation stage due to their direct role in lipid biosynthesis (Table 3). In this study, as NaNO_3_ supplementation engendered the highest growth and lipid accumulation, the activity of these lipogenic enzymes except CS correlates with our above results (Table 3).

**Table 3:** Comparison of the activity of key enzymes (nmol/min mg protein) related to lipid accumulation in *C. Cohnii* at three growth stages under different N-sources

N-sources	Growth stages	Activity of key enzymes (nmol/min mg protein)						
		FAS	ME	ACL	G6PD	NADP-ICDH	CS	NAD-ICDH
(NH_4_)_2_SO_4_	Growth	129.1 ± 4.2	40.5 ± 2.9	27.9 ± 6.2	252.1 ± 3.7	498.1 ± 1.9	1613.4 ± 21.6	248.4 ± 2.1
	Lipid accumulation	138.6 ± 1.9	22.5 ± 6.5	52.2 ± 7.3	238.9 ± 1.6	417.3 ± 3.7	1428.1 ± 13.2	235.9 ± 15
	Lipid turnover	125.9 ± 3.8	17.9 ± 2.4	18.6 ± 1.7	113.7 ± 6.1	331.6 ± 1.6	1341.7 ± 22.7	218.5 ± 2.2
NH_4_ HCO_3_	Growth	127.6 ± 6.1	41.5 ± 4.1	26.5 ± 2.5	253.7 ± 2.4	495.9 ± 3.9	1602.5 ± 31.8	247.5 ± 2.5
	Lipid accumulation	138.2 ± 2.4	24.7 ± 3.8	52.4 ± 1.8	242.1 ± 1.8	407.2 ± 5.2	1398.7 ± 21.9	231.7 ± 5.2
	Lipid turnover	125.5 ± 7.2	17.2 ± 3.4	19.1 ± 3.2	113.2 ± 2.5	328.6 ± 1.5	1356.2 ± 43.5	213.6 ± 9.2
(NH_2_)_2_CO	Growth	140.2 ± 2.7	45.7 ± 3.6	34.4 ± 1.9	241.5 ± 2.8	382.4 ± 7.4	1705.5 ± 23.38	198.5 ± 1.5
	Lipid accumulation	146.7 ± 11.9	34.2 ± 5.2	59.1 ± 2.8	239.3 ± 4.6	349.5 ± 1.8	1581.6 ± 32.8	194.6 ± 3.5
	Lipid turnover	130.9 ± 4.05	21.5 ± 1.9	22.2 ± 1.6	207.5 ± 1.9	364.9 ± 2.6	1520.5 ± 45.2	189.1 ± 1.5
NaNO_3_	Growth	141.9 ± 7.1	56.9 ± 6.5	40.5 ± 2.5	469.4 ± 1.2	253.9 ± 2.7	1491.4 ± 22.9	127.5 ± 3.1
	Lipid accumulation	153.5 ± 5.9	41.7 ± 7.5	72.8 ± 3.5	461.9 ± 3.6	168.6 ± 8.9	1325.7 ± 21.7	82.8 ± 1.5
	Lipid turnover	139.2 ± 5.6	29.4 ± 4.7	36.6 ± 4.5	331.8 ± 3.8	159.1 ± 3.4	1258.2 ± 12.8	103.4 ± 1.5

All experiments were performed in triplicate. The data presented here is mean ± SD

*G6PD* glucose-6-phosphate dehydrogenase, *CS*: citrate synthase, *NAD* ^*+*^ *-ICDH* NAD dependent isocitrate dehydrogenase, *NADP* ^*+*^ *-ICDH* NADP dependent isocitrate dehydrogenase, *ACL* ATP-citrate lyase, *ME* malic enzyme, *FAS* fatty acid synthase

**Table 4:** Comparison of the activity of key enzymes (nmol/min mg protein) related to lipid accumulation in *C. Cohnii* at three growth stages under different N-sources

NaNO_3_ conc.	Growth phase	Activity of key enzymes (nmol/min mg protein)						
		ME	ACL	FAS	G6PD	NADP-ICDH	CS	NAD-ICDH
0.2 g/L	Growth	58.7 ± 4.7	47.5 ± 4.7	152.1 ± 1.8	552.4 ± 1.5	168.4 ± 3.7	1483.8 ± 41.8	121.5 ± 2.9
	Lipid accumulation	59.1 ± 7.2	85.9 ± 2.8	168.2 ± 5.7	638.5 ± 2.4	87.7 ± 6.8	1428.1 ± 22.5	58.1 ± 1.8
	Lipid turnover	29.5 ± 3.9	43.6 ± 3.9	128.3 ± 9.5	413.1 ± 3.7	71.6 ± 2.9	1344.5 ± 13.9	53.9 ± 2.5
0.4 g/L	Growth	56.9 ± 6.5	40.5 ± 2.5	141.9 ± 2.1	469.4 ± 1.2	253.9 ± 2.71	1491.4 ± 22.5	127.5 ± 3.6
	Lipid accumulation	41.7 ± 7.5	72.8 ± 3.1	153.5 ± 5.9	461.9 ± 3.6	168.6 ± 8.9	1325.7 ± 21.7	82.8 ± 1.5
	Lipid turnover	29.4 ± 4.7	36.6 ± 4.5	139.2 ± 5.6	331.8 ± 3.8	159.1 ± 3.4	1258.2 ± 12.3	103.4 ± 1.5
0.8 g/L	Growth	47.8 ± 8.5	30.8 ± 2.4	146.8 ± 3.9	421.8 ± 1.8	264.9 ± 4.9	1505.9 ± 13.1	135.3 ± 2.8
	Lipid accumulation	35.9 ± 5.4	62.1 ± 1.9	146.9 ± 4.2	471.5 ± 1.2	269.5 ± 9.5	1381.3 ± 21.7	129.7 ± 1.5
	Lipid turnover	23.2 ± 2.9	38.5 ± 1.9	125.2 ± 6.3	398.1 ± 1.6	266.2 ± 1.6	1350.5 ± 31.4	126.2 ± 4.7
1.2 g/L	Growth	39.9 ± 2.6	28.9 ± 3.5	139.5 ± 2.1	413.6 ± 3.1	253.9 ± 2.6	1479.2 ± 25.5	131.5 ± 3.9
	Lipid accumulation	31.7 ± 4.7	56.2 ± 7.4	141.5 ± 2.5	385.9 ± 2.6	168.6 ± 2.5	1415.5 ± 42.2	129.8 ± 1.9
	Lipid turnover	27.5 ± 1.2	33.5 ± 5.2	133.9 ± 5.5	372.4 ± 1.4	159.1 ± 10.8	1378.4 ± 23.2	127.9 ± 4.5
1.6 g/L	Growth	38.5 ± 2.8	26.5 ± 2.8	132.7 ± 2.5	292.7 ± 1.8	253.9 ± 1.8	1461.4 ± 31.5	128.6 ± 2.5
	Lipid accumulation	33.8 ± 3.9	51.1 ± 2.4	127.9 ± 1.8	264.1 ± 2.5	168.6 ± 8.7	1409.7 ± 23.3	117.1 ± 2.5
	Lipid turnover	27.1 ± 6.5	24.9 ± 2.71	130.5 ± 1.6	227.5 ± 3.2	159.1 ± 3.7	1388.5 ± 12.5	117.9 ± 3.8

All experiments were performed in triplicate. The data presented here is mean ± SD

*G6PD* glucose-6-phosphate dehydrogenase, *CS* citrate synthase, *NAD* ^*+*^ *-ICDH* NAD dependent isocitrate dehydrogenase, *NADP* ^*+*^ *-ICDH* NADP dependent isocitrate dehydrogenase, *ACL* ATP-citrate lyase, *ME* malic enzyme, *FAS* fatty acid synthase

In present study, ACL activity was higher under NaNO_3_ than other N-sources which coincide with higher lipid production by providing more acetyl-CoA as substrate. While, (NH_4_)_2_SO_4_ showed the lowest lipid accumulation, exhibit reduced ACL activity by 30% as compared to NaNO_3_. Similar tendency was observed for FAS which catalyze the fatty acid synthesis and thus, its activity directly associate with lipid accumulation. Our results also showed that ACL and FAS activities were inversely correlate with NaNO_3_ concentrations in medium as both were most active in lipid accumulation stage at N-starved conditions (85.9 ± 2.8 and 168.2 ± 5.5 nmol/min mg protein, respectively) which were 58 and 31.5% higher than 1.6 g/L NaNO_3_ (51.1 ± 2.4, 127.9 ± 1.8 nmol/min mg protein, respectively) (Table 4). The activities of ME and G6PDH were higher in NaNO_3_ than (NH_4_)_2_SO_4_ culture, which were 40 and 86% higher in growth stage; 85 and 93% higher in lipid accumulation stage, respectively (Table 3).

NADP^+^-ICDH activity was significantly decreased from cell growth stage to lipid accumulation stage in all treatments (P < 0.05). In lipid accumulation stage, NaNO_3_ showed 147% low NADP^+^-ICDH activity (168.6 ± 8.9 nmol/min mg protein) than (NH_4_)_2_SO_4_ (417.3 ± 3.1 nmol/min mg protein). Correspondingly, NADP^+^-ICDH was less active in lower nitrate concentrations than higher (Table 4). In contrast, NAD^+^-ICDH activity was remained consistent throughout the cultivation period under respective nitrogen treatments except NaNO_3_. This indicated that N in the form of ammonium is preferred over nitrate and somehow still present in the culture medium during the entire growth (Table 3, *P* < 0.05). However, in case of NaNO_3_, a considerable decline (53.9%) was observed from growth stage to lipid accumulation stage perhaps due to unavailability of N in the form of ammonium. Alternatively, when comparing different N-sources, the activity of NAD^+^-ICDH in NaNO_3_ supplemented culture was significantly lower (100%) than that in (NH_4_)_2_SO_4_. Nevertheless, NAD^+^-ICDH activity increased radically with increase in NaNO_3_ concentration from 0.2 to 0.8 g/L and then a slight decrease in 1.2 and 1.6 g/L was observed (Table 4).

## Discussion

Lipid accumulation in oleaginous microorganisms is a dynamic process which depends on the growth conditions (like nutrients, temperature, pH, aeration and light in autotrophs) and growth stages. Therefore, for efficient lipid production, a proper selection of culture conditions and harvesting time are essential. The most commonly reported factor is nitrogen which showed significant effect on growth and lipid accumulation of in different microalgae (Ikaran et al. 2015; Khan et al. 2017; Lin and Lin 2011; Lin et al. 2017; Liu et al. 2016a, b; Portugal-Nunes et al. 2017). In present work we used different nitrogen sources and concentrations to improve algal growth, lipid production and especially DHA production. Our results prove that *C. cohnii* grow better on nitrate (NaNO_3_) than other ammonium or urea sources. Similar results were found in *Scenedesmus bijugatus* when cultured on six different N-sources over 18 days in which NaNO_3_ had shown better growth over other N-sources (Arumugam et al. 2013). In contrast, *S. rubescens* grow faster under ammonium than other nitrogen source treatments including urea during first 5 days of culture (Lin and Lin 2011).

It has been documented that high lipid content is usually accompanied by lower growth rates under different stresses which often lead to decreased biomass and hence overall lipid productivity (Dhup and Dhawan 2014; Mandotra et al. 2016). However as lipid biosynthesis in *C. cohnii* does not follow this usual pattern, and continue to accumulate regardless of LN or HN. Ultimately, highest lipid and DHA productivity was attained with NaNO_3_ supplementation. In another report, highest biomass productivity was obtained in *S. rubescens* when treated with urea and NaNO_3_ mixture while highest lipid productivity was gained with ammonium treatment (Lin and Lin 2011). Similarly, *T. pseudonana* (Griffiths and Harrison 2009), *N. oleoabundans* (Li et al. 2008), *S. costatum* (Rodolfi et al. 2008) and *S. dimorphous* (Benider et al. 2001) also significantly respond to different nitrogen sources. In another report, the highest biomass (5.03 g/L), TFA (24.9% DCW) and DHA contents (82.8 mg/g) of *Crypthecodinium* sp. SUN were achieved at 96 h of cultivation under light conditions (Sun et al. 2017). Under sesamol supplementation, highest biomass (3.9 g/L), TFA (21% DCW), DHA (41.3% TFA) and DHA productivity (58 mg/L day) was attained in *C. cohnii* ATCC 30556 (Liu et al. 2015). Pleissner and Eriksen (2012) reported highest biomass (2.1 g/L), TFA (111 mg/g) and DHA contents (36.2% TFA) in *C. cohnii* CCMP 316 when cultured on acetic acid as major carbon source. In the present study highest biomass (23.7 g/L), TFA (26.9% DCW), DHA (0.99 g/L) and DHA productivity (236 mg/L day) was attained in *C. cohnii* ATCC 30555 under NaNO_3_ supplementation. These results indicated that there is still much room to enhance lipid (% DCW) and DHA (% TFA) content in *C. cohnii* ATCC 30555.

Due to substrates depletion in lipid turnover stage, algae survived on subsequent expenditure of the reserved lipids. In fact, reserved lipid turnover commonly happened after transition from carbon excess to carbon starved conditions (Chang et al. 2013). Afterwards, net lipid productivity and lipid yield also declined. Collectively, biomass and lipid productivity confirmed a clear categorization of three different growth stages and our results present first report on identifying the three growth stages in *C. Cohnii* during the entire cultivation time. Thus, it can be concluded that most of the lipids were accumulated between 48 and 120 h.

It is assumed that when N is limited in the medium, proteins and other N-rich compounds are broken and used as nitrogen cell reservoir to support time-restricted growth processes (Ikaran et al. 2015). Thus, the recycling of cellular components could explain the biomass increase occurred in LN treatments. However, low final biomass content drastically reduced the overall productivity of the cell. On the other hand, higher nitrogen concentrations become toxic for algal survival. As previously stated, this is because of increased nitrate reductase activity at higher concentrations of NaNO_3_ leading to enhanced production of nitrite and ammonia that are accumulated in vivo (Dhup and Dhawan 2014). These accumulated nitrites and ammonia might act as toxins, resulting in decreased biomass production. Therefore, for higher biomass and lipid productivity can only achieved at MN concentration. These results were in consistent with the finding of Kim et al. (2016) and Lin and Lin (2011) that high biomass productivity can be attained at optimum N concentrations.

Our results also indicate that N-source greatly affects DHA content of *C. cohnii*; however, there was no significant influence on C16-C18 content. The content of C16–C18 series of *C. cohnii* was significantly (*P* < 0.05) lower as compared to *Chlorella vulgaris* (Converti et al. 2009) *Haematococcus pluvialis* (Damiani et al. 2010) and *S. rubescens* (Lin and Lin 2011). As, these algal species are used for biodiesel production, it was concluded that N-source might not be very important for biodiesel production in *C. cohnii*.

We report, for the first time, the activity of key enzymes potentially involved in lipid accumulation of *C. cohnii* under the influence of different N-sources. Most probably, the gradual decline in the activities of G6PDH, ME, NADP^+^-ICDH, CS and NAD^+^-ICDH during cultivation time was triggered by substrate depletion particularly glucose in the culture. The activity of FAS, ME, ACL and G6PDH were significantly (*P* < 0.05) higher under NaNO_3_ than other N-sources while NADP^+^-ICDH and NAD^+^-ICDH were least active. The provision of acetyl-CoA as an essential precursor and NADPH as reducing power source are essential for lipid biosynthesis in oleaginous microorganisms. As ACL is the key enzyme involved in citrate lyses and generates Acetyl-CoA in cytoplasm, the activity of ACL is correlated with specific rate of lipid biosynthesis. Therefore, ACL is higher under NaNO_3_ than other N-sources. Similar results were found in *Cunninghamella* sp. 2A1 (Hamid et al. 2011). In contrast, the ACL gene expression in *C. vulgaris* var L3 was below the detection level at 120–144 h of cultivation in N-starved cells as compared to N-replete conditions (Ikaran et al. 2015).

Two key enzymes, ME and G6PD, were usually known to provide NADPH for lipid biosynthesis (Ren et al. 2013). Previously, numerous studies have elucidated the role of ME in NADPH supply via conversion of malate to pyruvate and proposed as rate-limiting factor for fatty acid biosynthesis (Hao et al. 2014; Li et al. 2013; Liu et al. 2013; Wynn et al. 1999). Ratledge (2014) suggested that ME cannot provide all the required NADPH for lipid biosynthesis. Therefore, other enzymes including G6PDH and NADP^+^-ICDH (NADPH dependent ICDH coupled with pentose phosphate pathway (PPP) reaction might also be responsible for NADPH supply (Fig. 6). The higher activities of ME and G6PDH under NaNO_3_ than (NH_4_)_2_SO_4_ supplemented culture indicated that both enzymes are actively involved in lipid accumulation. Our results also indicated that G6PDH contributes more NADPH then ME in *C. cohnii* possibly through the following reactions:

$${\text{Glucose-}}6{\text{-phosphate}} + {\text{NADP}}^{ + } \to 6{\text{-phosphate-}}{\sc{d}}{\text{-glucono-}}1{,}5{\text{-lactone}} + {\text{NADPH}}$$

The role of G6PDH in NADPH supply for lipid biosynthesis was also reported in another oleaginous microalga *Chlorella protothecoides* (Xiong et al. 2010) and yeast *Yarrowia lipolytica* (Wasylenko et al. 2015). This could also suggest the involvement of G6PDH in lipogenic pathway is according to metabolic control theory that physiological changes in metabolic flux need equal changes of activity of all or many of the enzymes of pathway. Otherwise, G6PDH and ME together play a dual role in offering NADPH for lipid biosynthesis. This concept of ME together with G6PD in offering NADPH for lipogenesis might be novel in microalgae and needed to be explored.

Another NADPH-generating enzyme ICDH, present in cytosol (NADP^+^-ICDH) and mitochondria (NAD^+^-ICDH), cytosolic form of which contributes NADPH for lipid biosynthesis in some oleaginous microorganism (Tang et al. 2014). While, mitochondrial form is critically involved in regulating the intracellular carbon flow between TCA cycle and de novo lipid biogenesis pathway (Ratledge 2014). However localization of these enzymes is still unclear in *C. cohnii*. Our results suggested that ACL, G6PD, ME, NADP^+^-ICD were directly associated with increased lipid accumulation, the latter three were supposed to provide reducing power (NADPH) for FAS activity. It has previously been suggested that AMP is required for activation of NAD^+^-ICDH (Tang et al. 2015). When N starts depleting in the cell, AMP is deaminated by AMP deaminase to release ammonium and IMP which in turn down-regulates NAD^+^-ICDH activity and results in slowdown of carbon flow through TCA cycle (Fig. 6). This sequence of biochemical events creates an equilibration between isocitrate and citrate which is, later, transported to cytosol from mitochondria and subsequently cleaved by ACL into acetyl-CoA.

Furthermore, new cells proliferation discontinuation caused by N-depletion leads to termination of structural lipid biosynthesis. However, old cells continued to assimilate carbon source (glucose) and diverted into storage lipids, eventually accelerate total lipid production in *C. cohnii*. Similar results were found in *Scenedesmus rubescens* (Lin and Lin 2011), *Schizochytrium* sp. S31 (Chang et al. 2013), C. *Vulgaris* var. L3 (Ikaran et al. 2015), *Nannochloropsis salina* (Fakhry and El Maghraby 2015) and *Chlorella* sp. (Ördög et al. 2016). Conclusively, lipogenic enzymes ACL, G6PD, ME, NADP^+^-ICD and NAD^+^-ICDH, were shown to be vital in fatty acid biosynthesis of *C. cohnii*. The former three were up-regulated while later two were down-regulated in high lipid accumulation conditions via down-regulating glycolytic pathway and channelling carbon flux to elevated lipid production; when NaNO_3_ was provided as N-source at lower concentration.

In conclusion, lipid accumulation in oleaginous microorganisms is a dynamic process which depends on the growth conditions and growth phases. The present study showed that N-source and concentrations have great influence on growth and lipid accumulation. Three-way ANOVA also revealed significant differences between N-source, N-concentration and time for biomass (g/L) and total lipid content (g/L) in the culture of *C. cohnii* (*P* < 0.05; data not shown). Growth of *C. cohnii* was categorized in three distinct stages. N-concentrations did not influence lipid and DHA content of *C. cohnii*, however, for higher productivity optimal N-concentration (0.8 g/L NaNO_3_) proven to be the best. Therefore, lipid accumulation in *C. cohnii* could be considered as growth-associated process in stipulations of overall productivity. Presence of more than 50–60% of C16–C18 content in TFA suggested that *C. cohnii* can also be considered as potential source for biodiesel production. Lipogenic enzymes; ACL, G6PD, ME, NADP-ICD and NAD^+^-ICDH, were significantly responsive to N in growth stages and vital in fatty acid biosynthesis of *C. cohnii*. G6PDH coupled with pentose phosphate pathway (PPP), ME and/or ICDH (NADPH dependent) reaction were responsible for NADPH supply for lipid biosynthesis. This information will provide new research directions for lipid and DHA enhancement in *C. cohnii* in less time and cost effective manner.

## Abbreviations

DCW: dry cell weight
TL: total lipid
TFA: total fatty acid
DHA: docosahexaenoic acid
LN: low nitrogen
MN: medium nitrogen
HN: high nitrogen
FAS: fatty acid synthase
ME: malic enzyme
ACL: ATP:citrate lyase
G6PDH: glucose-6-phosphate dehydrogenase
CS: citrate synthase
NADP^+^-ICDH: NADP^+^-dependent isocitrate dehydrogenase
NAD^+^-ICDH: NAD^+^-dependent isocitrate dehydrogenase
PPP: pentose phosphate pathway
AMP: adenosine monophosphate
IMP: inosine monophosphate
